# Polymer Composites Filled with Metal Derivatives: A Review of Flame Retardants

**DOI:** 10.3390/polym13111701

**Published:** 2021-05-23

**Authors:** R. A. Ilyas, S. M. Sapuan, M. R. M. Asyraf, D. A. Z. N. Dayana, J. J. N. Amelia, M. S. A. Rani, Mohd Nor Faiz Norrrahim, N. M. Nurazzi, H. A. Aisyah, Shubham Sharma, M. R. Ishak, M. Rafidah, M. R. Razman

**Affiliations:** 1 School of Chemical and Energy Engineering, Faculty of Engineering, Universiti Teknologi Malaysia (UTM), Johor Bahru 81310, Johor, Malaysia; 2 Centre for Advanced Composite Materials (CACM), Universiti Teknologi Malaysia (UTM), Johor Bahru 81310, Johor, Malaysia; 3 Laboratory of Biocomposite Technology, Institute of Tropical Forestry and Forest Products (INTROP), Universiti Putra Malaysia (UPM), Serdang 43400, Selangor, Malaysia; sapuan@upm.edu.my; 4 Advanced Engineering Materials and Composites (AEMC), Department of Mechanical and Manufacturing Engineering, Faculty of Engineering, Universiti Putra Malaysia (UPM), Serdang 43400, Selangor, Malaysia; nuruldayanaa64@gmail.com (D.A.Z.N.D.); ameliajuria96@gmail.com (J.J.N.A.); 5 Department of Aerospace Engineering, Faculty of Engineering, Universiti Putra Malaysia (UPM), Serdang 43400, Selangor, Malaysia; asyrafriz96@gmail.com (M.R.M.A.); mohdridzwan@upm.edu.my (M.R.I.); 6 Solar Energy Research Institute (SERI), Universiti Kebangsaan Malaysia (UKM), Bangi 43600, Selangor, Malaysia; saifulasmal@gmail.com; 7 Centre for Tropicalisation, National Defence University of Malaysia, Kem Sungai Besi, Kuala Lumpur 57000, Malaysia; 8 Research Center for Chemical Defence, Universiti Pertahanan Nasional Malaysia (UPNM), Kem Perdana Sungai Besi, Kuala Lumpur 57000, Malaysia; faiznorrrahim@gmail.com; 9 Centre for Defence Foundation Studies, Universiti Pertahanan Nasional Malaysia (UPNM), Kem Perdana Sungai Besi, Kuala Lumpur 57000, Malaysia; mohd.nurazzi@gmail.com; 10 Department of Mechanical Engineering, Main Campus, IK Gujral Punjab Technical University, Kapurthala 144603, India; shubham543sharma@gmail.com or; 11 Department of Civil Engineering, Faculty of Engineering, Universiti Putra Malaysia (UPM), Serdang 43400, Selangor, Malaysia; rafidahmazlan16@gmail.com; 12 Research Centre for Sustainability Science and Governance (SGK), Institute for Environment and Development (LESTARI), Universiti Kebangsaan Malaysia (UKM), Bangi 43600, Selangor, Malaysia

**Keywords:** flame retardant, polymer composites, metal, metal components, characterization, combustion mechanism

## Abstract

Polymer composites filled with metal derivatives have been widely used in recent years, particularly as flame retardants, due to their superior characteristics, including high thermal behavior, low environmental degradation, and good fire resistance. The hybridization of metal and polymer composites produces various favorable properties, making them ideal materials for various advanced applications. The fire resistance performance of polymer composites can be enhanced by increasing the combustion capability of composite materials through the inclusion of metallic fireproof materials to protect the composites. The final properties of the metal-filled thermoplastic composites depend on several factors, including pore shape and distribution and morphology of metal particles. For example, fire safety equipment uses polyester thermoplastic and antimony sources with halogenated additives. The use of metals as additives in composites has captured the attention of researchers worldwide due to safety concern in consideration of people’s life and public properties. This review establishes the state-of-art flame resistance properties of metals/polymer composites for numerous industrial applications.

## 1. Introduction

**Figure 1:**
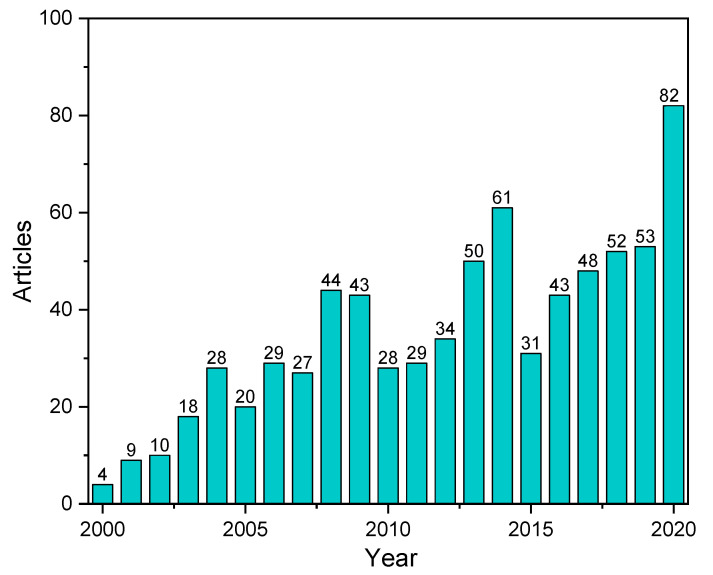
The number of publications on flame retardant metal polymer composite in the last two decades indicating the increasing interest from (Scopus, May 2021, Search: “Metal” and “Flame” and “Polymer”).

Polymer composites are globally recognized due to their thermal insulation properties. To improve their thermal and heat resistant performance further, certain metallic materials are added to polymers, such as copper [1], nickel [2], magnesium [3,4,5], and zinc [6]. The inclusion of metal component in polymer-based composites has produced new promising materials with high potential in various engineering sectors. Metal-filled composites offer numerous advantages, such as heat conduction, static electricity discharge, conversion of mechanical signal into electrical signal, and electromagnetic interference shielding signal [7]. Metal-filled polymer composites have been widely used in recent years, particularly as flame retardants, due to their superior characteristics. Figure 1 shows the trending of research conducted on flame retardant metal filled polymer composite. It can be seen that the trending of this research is increasing by 2000% over 20 years. These conductive polymer composites potentially combine significant advantageous characteristics of plastics and metals, offering less cost with high production rate [8], design flexibility [9], noncorrosive [10], and lightweight properties [11]. Processing methods, including the use of an internal mixer and extrusion and injection molding, can be adopted to fabricate these compounds [12,13,14].

Fiber reinforced composites can be classified into four groups according to their matrices: metal matrix composites (MMCs), ceramic matrix composites (CMCs), carbon/carbon composites (C/C), and polymer matrix composites (PMCs) or polymeric composites [15]. The four forms of polymer composite materials are widely used in vehicles, aircraft, spacecraft, boats [16], civil engineering [17], packing [18], cross arms in transmission towers [19], and portable fire extinguisher [20]. The use of polymer composites is increasing rapidly due to their excellent mechanical properties, such as creep [21,22,23], flexural [24], chemical resistance [18], and corrosion resistance [25]. Polymer materials are formed from hydrocarbon chains, which burn easily under intense heat; they can also burst into flames or emit smoke when exposed to light [26]. Numerous incidents have occurred previously in aircraft; at present, however, a remarkable increase in the fire tolerance of polymer composite materials can be observed during collisions [27]. To increase environmental sustainability, engineers and scientists are currently seeking to replace nonbiodegradable fibers (e.g., glass and carbon–aramids) with biodegradable fibers (e.g., corn [28],water hyacinth [29], coir [30], ginger [31,32], cotton [33,34], kenaf [11,35,36,37,38,39], sugarcane [40,41,42,43], flax [44], ramie [45], hemp [46], kapok [47], sisal [48], wood [17], oil palm [3,49], banana [50], and sugar palm [4,51,52,53,54,55,56,57,58,59,60]. However, polymer composites reinforced with natural fibers frequently heat up efficiently [61] and exhibit high thermal conductivity [62].

In this review, the term “flame retardant” (FR) is applied to various industrial and consumer products. The requirements of FRs that are relevant to the product’s quality and longevity must be fulfilled. FRs provide fire protection by restricting the flow of oxygen to the flames. Expanded polyurethane foams and their compound foams are frequently used in fire suppressants, increasing metal combustion temperature, and minimizing flame diffusion [63]. Meanwhile, other important features, such as mechanical and thermal performance and environmental friendliness, e.g., not posing hazards to humans and the environment and capable of being recycled and reused, must be maintained. The usage of halogenated materials to prepare FRs is an efficient process. However, a gradual decrease in the acceptability of these products has been noted due to the increasing emphasis on environmental and health issues involving FRs.

Numerous metal particles, such as aluminum, copper, zinc, stainless steel, silver, gold, and nickel, are used in different polymer matrices [64]. Metal-filled polymer composites are reported to have increased electrical and thermal conductivity. Table 1 lists the metallic fillers used in FR applications. In some polymer composites, the metal and natural fibers such as kenaf [65], flax [66], and jute [67] have been added together with the polymeric resin to enhance the structural and thermal stability. Krishnasamy et al. [65] reported that the addition of aluminum and copper in jute epoxy hybrid composite resulted in the excellent thermal stability, as well as improved in their mechanical strength such as tensile and flexural performance. According to El-sabbagh et al. [68], by adding some amount of magnesium hydroxide (Mg(OH)_2_)—about 20–30 wt% to the flax reinforced polypropylene composite improved the onset of decomposition temperature and LOI values. The composite comprising 50 wt% flax and 30 wt% flame retardant, in particular, achieved a 27% LOI score and a V-2 grade from the UL-94 test, with a long burning period and no dripping. By releasing a large amount of water, Mg(OH)_2_ have efficient flame retardant efficiency by diluting the amount of fuel required to support combustion. They also reported that the addition of fibers and Mg(OH)_2_ increased tensile stiffness in this study.

Thus, the importance of flame retardants of metal polymers composite is discussed in this review. The impact of various addition of metal component, such as zinc, copper, aluminum, and nickel, on the flammability and fire retardancy of polymer composites are examined, with an emphasis on natural fiber reinforced polymer composites. The method of combustion and the commonly employed flammability measurement methods for polymer-based composites are also discussed. Finally, this review aims to provide state-of-the-art views of the fire resistance performance of various metal filler–polymer composites.

## 2. Flame Retardancy of Polymer Reinforced Composites

**Figure 2:**
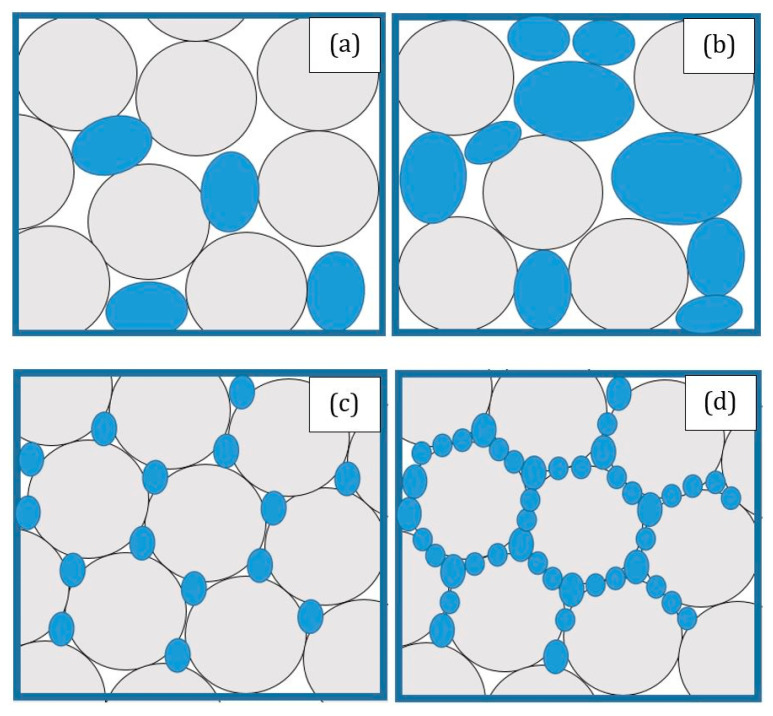
Illustration of random (**a**,**b**) and segregated (**c**,**d**) distributions of metal particles in polymer composites.

Using a mixture of recyclable waste polymer and PP fiber-reinforced materials has been demonstrated to be the most suitable method for the economical production of an entirely recyclable fire safety engineering design [70]. Figure 2 shows the metal particle distributions in polymer composites. In the case of natural fiber-reinforced polymer composites, their susceptibility to heat and flame retardant is one of the limitations. This is because of the presence of cellulose in plant fibers and hydrocarbon-based polymers causes the composites to be highly flammable [71]. Understanding the thermal decomposition and flammability of bio-based fibers, polymers, and their composites is crucial. Furthermore, appropriate flame retardant treatments and addition of metal component in these composites have been shown to significantly improve their thermal stability and fire resistance. According to Girisha et al. [72], adding amount of cellulosic fiber raises flammability of sisal/coir fiber reinforced epoxy composites since natural fiber embraces combustion. It is a weak flame retardant owing to the formation of a surface layer during the pyrolysis of the cellulosic material, which has a low fire resistance and act as a fire supporter, preventing heat from being transmitted to unpyrolized products.

**Figure 3:**
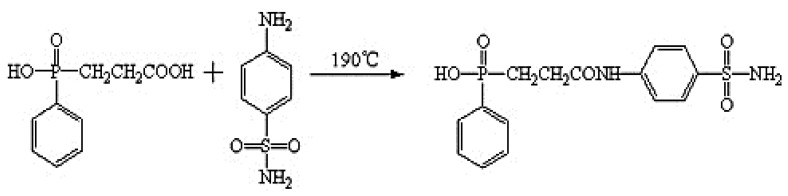
Chemical reaction of flame retardant synthesis of composites. Reproduced with copyright permission from Zhao et al. [73], Elsevier.

To date, several studies have investigated the flame retardancy of polymer composites filled with metal derivatives. Bar et al. [69] examined the flame retardation of polymer compounds. They determined that a particular fire retardation method and the effect of polymer composites were enhanced by FR with different composite properties. Figure 3 illustrates the FR synthesis process in composites via melt condensation reaction. Methodological approaches for improving the fireproofing effect are based on firefighting chemicals. The insertion of FR compounds or micro/nano FR fillers into a polymer backbone can increase the polymer matrix’s flame slope. To achieve a highly FR composite material, the polymer frame matrix must exhibit more than 15% fireproofing filtration; this condition compromises its mechanical properties. In the current analysis, halogen-based FRs can increase the flame retardation effect of the formulation at lower concentrations relative to metal hydroxides. However, halogen is toxic to the atmosphere, and thus, its use has been banned. The size of the nano type filer would be very harmful to human health.

Zadeh et al. [74] researched the recovery of polymerized FR mixtures enhanced with palm fiber. Their findings demonstrated that magnesium hydroxide (Mg(OH)_2_) is used on artificial composites; the material was fireproofed. This research was conducted using a conical calorimeter to measure fire resistance and analyze limiting oxygen index (LOI). The exhaust gas emission rate, overall heat release rate of the composite material comprising the refractory filler were tested in accordance with oxygen consumption theory. The most important development was that recycled materials and palm oil waste can produce composites that are affordable and environmentally safe. Although palm oil retains the mechanical properties of ternary composite mixtures, FRs exhibit heat resistance. This study showed that fire protection decreases mechanical efficiency. However, palm fiber increases the total strength of the construction material, helping achieve physical and mechanical properties. Composite products with 10% fibers and 1% binder (a combination of polyvinyl anhydride and maleic acid) exhibit mechanical strength and thermal tolerance.

Recently, the literature that presents the flame retardancy findings of metal composites has emerged. Yuan et al. [75] experimented on melamine (MA)-modified graphene oxide (GO). They found that slowing the combustion of PP altered MA-modified GO via heavy Δ–Δ interactions and hydrogen and electrostatic bonds. In their thesis, GO used the modified Hummers process to oxidize graphite powder. In particular, 0.6 g of GO and 3 g of MA were combined to create FGO. Interestingly, the findings of the transmission electron microscopy (TEM) and scanning electron microscopy (SEM) demonstrated that FGO nanosheets are evenly scattered in polymer matrices with embedded and flaky microstructures. FGO/PP nanocomposites exhibited better thermal stability and flame tolerance relative to their GO counterparts.

Fiber metal laminates (FMLs) are often used in the manufacture of hybrid natural fiber/metal polymer composites because of their strong electrical and thermal conductivity. FMLs are lightweight construction structures made up of thin metal contrasting with thin composite plies of metal as exterior surfaces (0.3–0.5 mm in thickness). Rather than improved thermal properties, various studies on hybridization of natural fiber with FML has been reported to enhanced their mechanical properties. Because of the inclusion of aluminum layers in the composite structure, tensile, flexural, and impact properties in sisal fiber reinforced aluminum laminates is significantly improved [76]. The tensile strength and dimensional stability of kenaf woven fabric reinforced with polypropylene also improved when utilizing FML aluminum, as reported by Ishak et al. [77].

## 3. Combustion Mechanism and Flame Retardancy of Composites

**Figure 4:**
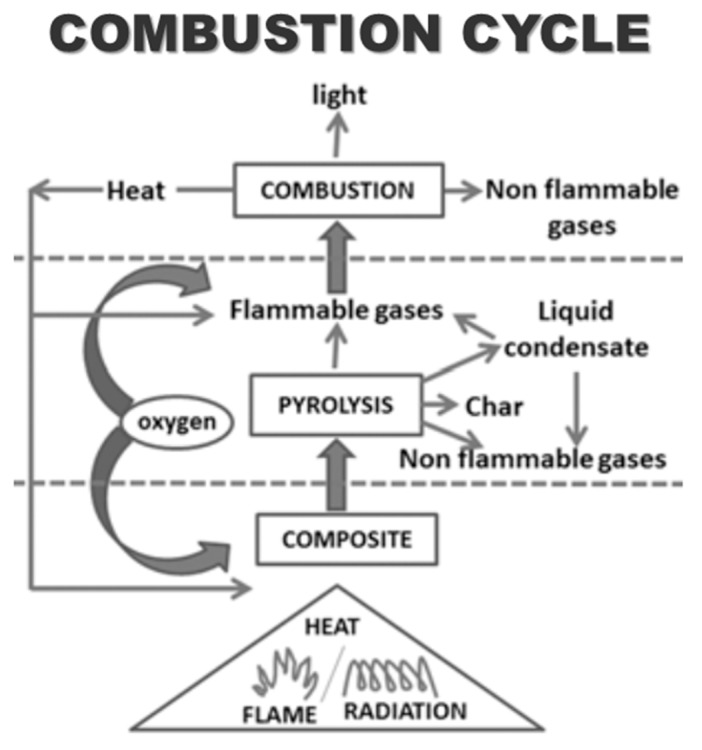
Flame retardancy approach and combustion cycle. Reproduced with copyright permission from Bar et al. [73], Springer.

Considering the continuous increase in plastic waste and environmental degradation, biodegradables that use renewable products as a substitute for traditional petroleum plastics are becoming increasingly common. In recent years, halogen-free FRs have elicited considerable attention because halogen fuels used for combustion produce large and fast volumes of fires. Some types of fire prevention equipment include thermoplastic polyester and commonly use halogenation agents, special bromine, polymers, and antimony sources. Halogen-free systems pose problems, some of which are new to municipal authorities; halogen-free phosphorus compounds, surface treatments, and reaction processes have been used in polyethylene terephthalate (PET) textiles for many years [78]. Figure 4 depicts the combustion cycle and potential flame retardancy approach. 

### 3.1. Combustion Mechanism

**Figure 5:**
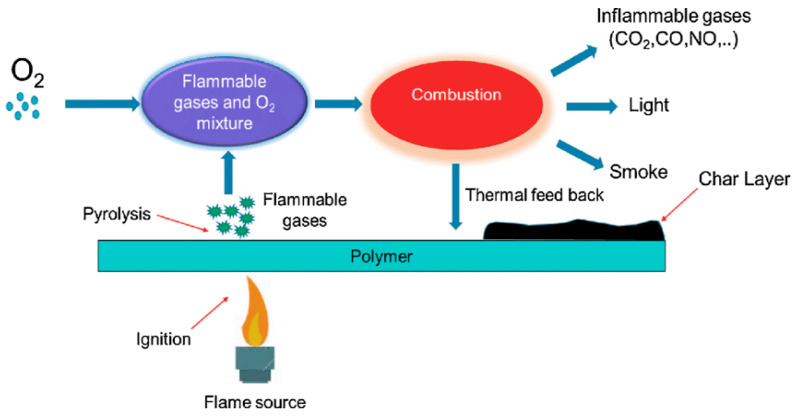
Schematic diagram for the combustion process cycle of polymers.

Several researchers have also investigated the burning of metals in the shapes of chains, rods, and ribbons. Figure 5 illustrates the combustion mechanism of polymers. A motion picture technique is used to calculate burning times in different atmospheres. The addition of small amounts of water vapor exhibits a remarkably significant effect. Effort has been exerted to quantify burning times on the basis of the fact that transport processes are considerably slower than chemical reactions, and thus, the pace of the burning phase can be controlled. Burn instability processes in functional combustion systems are highly complicated due to coupled correlations and intrinsic nonlinearities correlated with the involved phenomena. Consequently, most instability processes cannot be modelled or represented using conventional analytical techniques unless several simplification processes are introduced to solve the issue.

### 3.2. Flame Retardant Techniques

**Figure 6:**
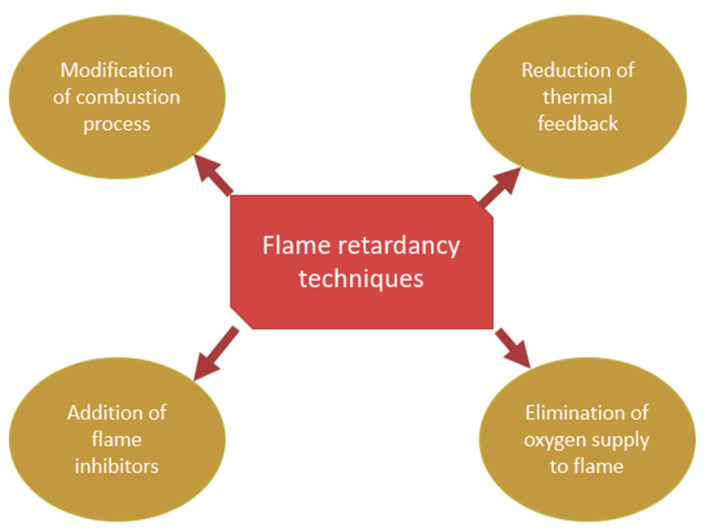
Methods of flame retardancy.

Advanced fire protection equipment may include three components: (1) acid source, including ammonium polyphosphate (APP); (2) Fourier transform infrared (FTIR) output (metals may increase the release of ammonia and carbon dioxide); and (3) fuel gas. Figure 6 illustrates flame retardancy techniques. The finding is consistent with microscale combustion calorimetry (MCC) and cone calorimetry (CONE) products. That is, if oxygen amounts can be diluted more easily, then ammonia and carbon dioxide emissions will increase. The reduced gas emissions can result in a decline in a material’s heat release. The preceding experimental results and previous experiments have been developed for potential fire safety methods involving metals (iron, magnesium, aluminum, and zinc) in paraffin/intumescent FR (IFR) systems. Applications may produce polyphosphoric acid at high temperatures, and polyphosphoric acid may react with the pentaerythritol OH group. The FRs filler would emit ammonia gas to suppress the oxygen. Simultaneously, polyphosphoric acid can interact with metal oxide (metal +), in which this structure may be extended to the stability of polyphosphoric acid, and increasing the molecular weight of polyphosphoric acid can increase the viscosity of the FR layer, making the protective layer more efficient in shielding the polymer matrix [79].

One of the disadvantages of polymer composites is their high flammability, which prevents their use in a variety of areas. As a result, improving their flame retardant properties is important, and a lot of effort has gone into it. The most effective approach for modifying the flame retardant properties is to incorporate FRs during the compounding phase. Recent findings show that the development in flame-retardant additives has been developed rapidly and new trends were discovered. The use of Ammonium polyphosphate (APP) in order to boost the flame retardant properties has found to increase the fire properties in polymer-based composites [80,81]. In addition, a combination of APP with other flame retardants, such as expandable graphite, SiO_2_, or CaCO_3_ [82,83,84], recorded to increase the effectiveness of fire retardancy. Furthermore, there are a variety of inorganic additives [85], organic flame retardants, nano-fillers [86], and anionic nano-clays [87] that were reported can improve the flame retardancy.

## 4. Characterization of Composites after Flame Retardant Treatment

The fire efficiency of the reinforcement components is enhanced by treating with FR chemicals. However, the fireproofing performance of the polymer matrix can be increased by using micro/nano FR fillers or by adding FR composites to the polymer backbone. This process resolves the degradation of micro-compounds and nanofillers used in newly developed polymers and polymer replacements, along with their effects on composite features, such as automatic and thermal effects. However, the production of all FR material composites is the same during the development stage; for further innovations, researchers must focus on the production of healthy and environmentally sustainable FRs that can increase the firing efficiency of composite products at minimal concentration levels [69]. Table 2 summarizes metal particle functions as FR additives in various composites.

Interests in the flame retardancy of metal composites have been renewed recently. Kusakli et al. [110] improved the flame retardancy and mechanical properties of epoxy composites by using FR with red mud (RM) waste to demonstrate the FR properties of these polymer composites and to prove that FR systems are safe to use because of their high chemical and thermal resistance. The effects of ammonium tetrafluoroborate (ATFB), RM waste and aluminum hydroxide (Al(OH)_3_) on the composites’ mechanical and flame-resistant characteristics were investigated. RM waste was ground and sieved into particles measuring less than 63 μm to prepare the ER-based composite materials. Subsequently, different amounts of ATFB, RM waste, and Al(OH)_3_ were mixed with the ER matrix at 2000 rpm via mechanical stirring and ultrasonication for 1.5 h at 60 °C to achieve strong dispersion. The combustion test demonstrated that the RM–ATFB–Al(OH)_3_ mixture can be efficient if halogen-free FR is used in coating and construction areas for materials based on epoxy. Al_2_O_3_, which is formed by Al(OH)_3_ decomposition reaction, prevents heat and oxygen from being transferred between the material and the environment, and thus, additional oxygen is required to ignite the sample. This previous study indicated that the burned area of the composite was only a small proportion of the total. For this composite, the experimental and estimated LOI values were 26 and 29, respectively. Burning studies were conducted to test the flammability of hydroxide and boron retardants.

The extensive literature signifies that numerous studies have examined the flame retardancy of metal composites. Song et al. [111] investigated the effect of metal chelates on flame retardancy of polypropylene (PP)/PDBPP. This study demonstrated the synthesis of the new oligomeric phosphorus-nitrogen containing intumescent flame retardant, poly(4,4-diamino diphenyl methane-O-bicydicpentaerythritol phosphate–phosphate) (PDBPP). Moreover, this study assessed whether the presence of metal chelates can enhance the flame retardancy of PP/PDBPP systems. Two metal chelates (zinc and chromium acetylacetone) are commercially available for the purpose of analysis. They were used as synergic agents without additional purification and other starting materials and solvents. The LOI value of PP/PDBPP (80/20) increased to 25, indicating a substantial improvement in PP flame retardancy in the presence of PDBPP. As demonstrated via Raman spectroscopy, infrared spectroscopy, and electron scanning microscopy, metal chelates (a decomposition product of PDBPP) may react with polyphosphoric acid as a cross-related network. A more compact layer, which produced PP/PDBPP with enhanced thermal and FR performance, was formed via salt bridges. This result showed that highly valuable metal chelates may improve the delays of FRs. Chang et al. [121] studied the flame retardancy and thermal stability of ethylene-vinyl acetate (EVA) copolymer nanocomposites when reinforced with alumina trihydrate (ATH) and montmorillonite (MMT). Organoclay (OMMT) was prepared by adding 20 g of MMT to 92 meq100 g^−1^ to 1000 mL of deionized (DI) water, with cationic exchange capacity. The mixture was agitated for 6 h and labelled as Solution A. Then, 4.96 g of octadecyl amine was dissolved in 50 mL of DI water, stirred for 3 h and called Solution B. Solutions A and B were mixed and heated for 3 h at 80 °C. OMMT was stored after 24 h of filtration, washing, and vacuum drying. The best FR quality (40/60%) of the total cable wire included a small amount of MMT. This study indicated that superior tensile strength was achieved at 3 wt% MMT. Furthermore, EVA’s flame retardancy is free from halogen, with 3% OMMT and 47% ATH achieving optimum deformation and flame resistance (LOI = 28). The tensile and fire inhibition characteristics of the nanocomposites were improved significantly.

Researchers have attempted to evaluate the effect of the flame retardancy of metal composites. Suppakarn and Jarukumjorn [124] examined the mechanical and thermal properties of sisal/PP composites and determined the effects of FR type and content. The objective of this research was to add FRs Mg(OH)_2_ and zinc borate (ZnB) to enhance the flame resistance of the morphological and mechanical features of sisal/PP composites. The ratio of Mg(OH)_2_ to ZnB was different in each sisal/PP composite location, while the overall content was maintained frequently at 30 wt%. Maleic anhydride grafted PP (MAPP) was also used as a compatibilizer to enhance adhesion between PP/sisal and PP/FRs. The flammability of PP and PP composites was investigated using ASTM D635 (standard test method for rate of burning and/or extent and time of burning of plastics in a horizontal position). The specimen was held horizontally, and a flame was applied to one end of the sample. Marking time was recorded from the first mark, 25 mm from the end of the mark to the second mark, and 100 mm from the end of the mark. Three specimens were tested for each composite. The composites were then measured for burning speed. The burning rate of the 30 ZnB composite was close to that of the clean PP. Meanwhile, the burning rate was immediately below that of pure PP for the 15 Mg/15 ZnB composite stage. Consequently, Mg(OH)_2_ more efficiently decreased PP composite’s burning intensity than ZnB addition. This study demonstrated comparable tensile and flexural properties with the addition of Mg(OH)_2_ and ZnB without FRs for the sisal/PP composites. The addition of Mg(OH)_2_ and ZnB enhanced the flame retardancy of sisal/PP composites without losing their mechanical properties.

The past 30 years have witnessed increasingly rapid advances in the flame retardancy of metal composites. Davies et al. [115] conducted a study on the sensitization of the heat treatment of APP by selected metal ions and their potential to improved cotton fabric flame retardancy. The effect of adding a series of metal salts on the thermal behavior of APP as a means of sensitizing FR behavior was determined. The addition of metal salts apparently improved the FR efficiency of APP as part of the pentaerythritol FR method in a PP matrix. Dry APP (MCM) mixtures were prepared from various dry mixtures of 2% w/w of each metal salt. Ferric sulphate APP mixtures with salt amounts ranging from 1% to 5% w/w were also prepared. Interestingly, the sodium and magnesium salts produce the highest increases with ΔLOI_(salt)_ ≥ 1.8. Salts, such as manganese and zinc sulphates, the largest of which existed at the same DTG transition temperatures, exhibited lower DLOI (salt) values of 0.9 × 10^1.1^. This study demonstrated that some metal ions, particularly Mn^2+^ and Zn^2+^, are absent when facilitating the thermal degradation of APP, improving the performance of flame retardation in the polymer at lower temperatures. The metal ion-doped APP did not only exhibit higher sensitization to cellulose decomposition in the presence of cellulose, but it also improved flame retardancy by limiting the oxygen index of cotton fabric.

Studies on composite materials have demonstrated the importance of the flame retardancy of metal composites. Beyer [103] investigated the fire-resistant property of EVA nanocomposites and advancements in the combination of nanofillers with ATH. FR nanocomposites were found to be formulated with modified layered silicates by melt blending ethylene-vinyl acetate (EVA) copolymers (MMT). Thermogravimetry (TGA) was conducted in various atmospheres, such as nitrogen and air. A major improvement in the thermal stability of the nanocomposites based on silicate was demonstrated. Moreover, a cone calorimeter was used to examine the fire properties of materials. The observation from the results showed a reduction in the cone calorimeter’s heat release peak, indicating that the char formation of the nanocomposites was enhanced and was responsible for the improved flame retardation. The thermal properties of EVA were reportedly improved. Moreover, EVA nanocomposites combined with metal hydroxides, such as ATH, presented the possibility of FRs as new compounds with reduced total filler contents.

The problem of metal-filled polymer composites flame retardancy has received considerable attention. Yen et al. [88] conducted research on the synergistic FR effects of metal hydroxides and nano-clay on EVA composites. The results of the observation indicated that LOI value was significantly improved when 1–2% weight nano-clay was replaced with aluminum hydroxide or Mg(OH)_2_ in the EVA blend, while maintaining the V–0 rating. The CONE test data showed that the peak heat release rate decrease was approximately 28% to 47%. Smoke density data registered a decrease of approximately 16–25%. TGA data also showed that the thermal stability and char residue of the EVA samples were improved by nano-clay. The metal oxide layer on the burning surface was also suggested to be reinforced by creating a silicate layer. Lujan-Acosta et al. [99] studied the synergistic effects of organo-modified MMT and metal hydroxides, namely, Mg(OH)_2_ and ATH, as FRs in low-density polyethylene (LDPE)/EVA nanocomposites integrated with amino alcohol. Grafted polyethylene was found to be compatible with LDPE/EVA/clay/FR nanocomposites (PEgDMAE). The structural characterization of nanocomposites was performed via X-ray diffraction (XRD) analysis and scanning transmission electron microscopy (STEM). In addition, horizontal burning and CONE tests for UL-94 and LOI were conducted to analyze the FR properties of nanocomposites. Thermal degradation output was also tested via FTIR coupled with TGA (TG-FTIR). The XRD analysis showed a change in the d001 plane to the lower-angle characteristic of the clay peak, indicating an intercalated–exfoliated microstructure. In the polymer matrix, which was expressed in FR properties, a significant dispersion of FRs by Mg(OH)_2_ and ATH was observed in the STEM images. Lujan-Acosta et al. [99] reported that the TG-FTIR result showed excellent thermal stability of the nanocomposites, and a major reduction was observed in the gases emitted during combustion. Therefore, the FR mechanism of LDPE/PEgDMAE/EVA/clay/Mg(OH)_2_ nanocomposite was proposed on the basis of the findings of thermal degradation and thermal stability.

Jeencham et al. [92] examined the effect of FRs on the mechanical and thermal properties of sisal fiber/PP composites. The FR performance of APP, Mg(OH)_2_, ZnB, and sisal fiber/PP composite combination was presented. The experiment was performed via vertical and horizontal burning tests. Moreover, MAPP was used as an integration enhancer for the PP/fiber and PP/FR systems. The result indicated that the addition of FRs to the composites decelerated the burning rate of the PP composite. Among several types of FRs, APP showed that the most powerful FR improvement was achieved by the PP composites during the vertical and horizontal burning tests. Jeencham et al. [92] reported that the flame retardancy and thermal stability of PP composites were enhanced without weakening their mechanical properties.

Li et al. [125] investigated the varying effects of flame retardancy and aluminum phosphonate (AlPi) mechanisms on poly (p-phenylene oxide) (PPO), thermoplastic polyurethane (TPU), and PP. The influence of AlPi on the flame retardancy of the three polymers (PPO, TPU, and PP) was determined. Experiments using LOI, SEM, vertical burning test (UL-94), CONE, and TGA were conducted. The results showed that the addition of AlPi substantially increased the LOI values of PPO and PP, but had nearly no effect on the LOI of TPU. In addition, although PP increased, the peak heat release rates of PPO and TPU decreased. A dense char layer was developed by the PPO composite, demonstrating the best flame retardancy. Meanwhile, a thinner char layer was developed by the TPU composite. During combustion, however, the PP composite did not form any char layer. The addition of AlPi effectively reduced the TPU matrix’s melt dripping and improved flame retardation. AlPi/PP composites acted as a fire resistor, decreasing the productive combustion heat of the volatiles, and increasing the amount of released carbon monoxide. Sharma and Saxena [105] studied the FR smoke suppressant protection provided by polyvinyl chloride (PVC). The metal-based organic (MBO) complexes were synthesized to be used as FR smoke suppressants in PVC formulations. FR smoke suppressant ingredients with 325–400 mesh size were mixed with a 2–5% thickener solution and appropriate amounts of wetting, anti-settling and anti-foaming agents. Vinyl acetate and vinyl versatate copolymer emulsions (binders) were modified by reacting with a polymeric plasticizer and dihydroxydimethylol ethylene urea. The observation results showed that the smoke suppression output achieved outstanding results when either of the two MBOs was used. Moreover, LOI increased, particularly when the PVC samples were plasticized using a phosphate plasticizer. The coated cables did not exhibit any surface flame spread when exposed. Moreover, the generation of smoke was extremely poor for the coated cables. The coatings were highly efficient in minimizing the burning actions of power cables, significantly improving circuit failure time.

The effect of FR ZnB or boric acid mixed with Mg(OH)_2_ was observed in Sain et al. [91] on the FR and mechanical properties of natural fiber/PP composites with Mg(OH)_2_. The experiment was conducted using the horizontal burning rate test. The specimen was held horizontally, and a flame ignited by gas was added to flare up the end of the specimen. In addition, LOI analysis was performed by placing the sample vertically in a glass chamber wherein nitrogen and oxygen flow was controlled. The observation results indicated that 25% of Mg(OH)_2_ can significantly minimize the filled composite’s flammability to approximately 50% without FR. The partial substitution of 5% Mg(OH)_2_ with ZnB or boric acid exhibited a retarding effect on the flame retardancy properties of Mg(OH)_2_. Mg(OH)_2_ can affect the flammability of natural fiber-filled PP composites by reducing the capability to ignite the composites. Even when Mg(OH)_2_ was used with ZnB and boric acid, no synergetic effect was observed. Finally, a small reduction in the mechanical properties of the composites was observed with the combination of FRs.

Braun et al. [97] investigated the fire retardancy mechanisms of metal phosphonates and metal phosphonates combined with MA cyanide (MC) in glass-fiber-reinforced poly (1,4-butylene terephthalate) (PBT/GF). The result showed the pyrolysis and fire activity of PBT/GF with two distinct metal phosphonates as fire retardants with and without MC. An experiment was performed via TGA and TGA coupled with infrared spectroscopy. The analysis data were collected from flammability tests, CONE tests, and SEM/energy-dispersive X-ray spectroscopy (EDS) and X-ray fluorescence (XRF) spectroscopy. Dosages of approximately 13% to 20% of halogen-free FR aluminum phosphonate or aluminum phosphonate with MC in PBT/GF were able to meet the requirements for electrical engineering and computer applications (UL 94 1⁄4 V–0; LOI > 42%). Meanwhile, the average 16% for zinc phosphonate with MC did not satisfactorily increase fire behavior (UL 94 ¼ HB; LOI ¼ 27–28%). The AlPi content indicated that the residue remained mechanically intact in the examined specimen and covered the polymeric materials from pyrolysis. This phenomenon created superior flame retardancy in the AlPi materials and met the application test criteria. Gallo et al. [102] studied the synergistic effects between nanometric metal oxides and phosphonate. They determined that for petroleum-based plastics, the FR synergy between phosphorus-based additives and metal oxides was used and applied to bio-based materials. The pyrolysis and flame retardancy properties of AlPi, along with antimony oxide and nanometric iron oxide, on a blend of poly (3-hydroxybutyrate-co-3-hydroxyvalerate)/poly(butyleneadipate-co-terephthalate) (PHBV/PBAT) were analyzed. Crystallinity changed, and the reaction between the polymer and additives may influence biodegradation because biodegradation occurred first in the unstructured polymer region. Moreover, AlPi decomposed separately from PHBV/Ecoflex, which was primarily in gas phase as phosphonic acid. In the solid phase, AlPi was partly retained as inorganic phosphate. However, the addition of metal oxide did not considerably affect the thermal and combustion activities of PHBV/Ecoflex. Only the synchronous inclusion of AlPi and metal oxide with a global filler content of 10 wt% contributed to the good effect on flame retardancy, increasing the value for UL 94 rating and inducing additional char formation. Fire retardation improvement was due to the increase in char production and the preferred changes in the classification of UL 94. Moreover, the nanofiller and phosphorus components worked together in the FR mechanism, with the primary mechanism behaving like FR in gas state.

**Figure 7:**
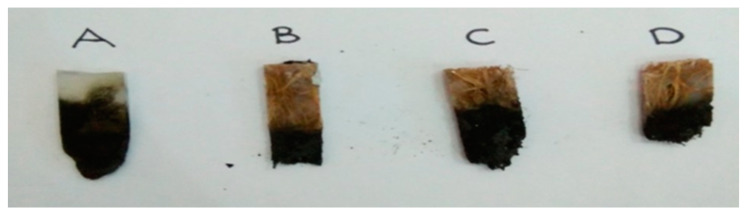
Sample of OPEFB/ PET yarn/magnesium hydroxide reinforced epoxy hybrid composites after the horizontal burning test. (**A**): 0% OPEFB; (**B**): 20% OPEFB; (**C**): 20% OPEFB; 35% OPEFB; and (**D**): 50% OPEFB. Reproduced with copyright permission from Suriani et al. [3], Polymers MDPI.

Xiao et al. [101] examined the effect of ionic liquid-based metal–organic hybrid on the thermal degradation, fire retardancy, and smoke suppression properties of ER composites. An anion exchange occurred between phosphomolybdic acid and phosphonate-based ionic liquid. A new multifunctional ionic liquid-based metal–organic mixture (PMAIL) was developed and applied to ER as an effective FR. PMAIL-based ER composite was prepared. Firstly, 1.24 g of PMAIL was dispersed into 15 mL of ethanol. Then, 100 g bisphenol A diglycidyl ether ER was added with magnetic stirring. Secondly, the mixture was stirred at 100 °C for 1 h to eliminate ethanol. Lastly, the mixture was cast into preheated molds and cured at 100 °C for 2 h. The carbonized yield of ER-PMAIL1 (1 wt% addition) composite at 700 °C was dramatically increased by 108% from 12% and 25% for ER. Meanwhile, ER-PMAIL6 (6 wt% addition) composite could reach V–0 rating in the UL-94 vertical burning experiment. The total smoke output and peak heat release rate of ER-PMAIL6 decreased by 15.4% and 31%, respectively, compared with ER. The carbonized yield of ER-PMAIL6 was improved by nearly 160% from 9% to 25% compared with ER for the CONE test, indicating strong mechanical properties and intumescent carbonized layer for superior flame retardance. Suriani et al. [3] investigated the horizontal burning rate by using Mg(OH)_2_ to determine its capability as FR composite. Different percentages of oil palm empty fruit bunch fiber (OPEFB) were added, with PET yarn and Mg(OH)_2_ as controls. The burning test showed that the specimen with 20% OPEFB exhibited better flammability properties, with the lowest average burning rate (11.47 mm/min). Figure 7 depicts the sample of specimens after the horizontal burning test. A conclusion was drawn that the flammability and tensile properties of OPEFB fiber-reinforced epoxy composites were reduced when fiber volume contents were increased at an optimal loading of 20%, with values of 11.47 mm/min and 4.29 MPa, respectively.

## 5. The Economic Analysis of Metal-Filled Polymer Composites

In the last few decades, there has been a surge of concern in the effect of metal materials on composite manufacturing particularly in thermal stability component. Using metal as a reinforcing medium has the ability to improve recycling rates while still locating high-value uses for polymer composites. For the case of polymer composites filled with metal derivatives, by eliminating the exploration, processing, and shipping, it will dramatically minimize environmental impacts. The latest study by Bulei et al. [126] found that recycle process of aluminum alloys for metal matrix composite has economic and technological advantages. It is also mention in this paper that it is not cost-effective to use fresh raw materials to produce high-value-added goods, where they can be obtained from scrap by competitive non-ferrous metal recycling technologies. In terms of waste utilization, the strategies adopted are achievable from an economic standpoint. As a result, the cost-benefit ratio satisfies the economic feasibility criterion. In addition, in a study by Dong et al. [127] concerning environmental effects modelling and the economic effects of composite recycling for fiber reinforced polymers, which motivated both environmental and economic factors in creating recycling routes for increasing quantity of fiber reinforced polymer scrap generated. Both glass and carbon fiber reinforced polymers recycling methods were compared to low-value end-of-life options, with pyrolysis appearing to be an appealing solution to recycling carbon fiber reinforced polymers that meets both environmental and economic benefits. However, in terms of cost, using metal as a reinforcement must be considered. Newest study on the evaluation and techno-economic analysis of the metal alloys tubes of multi-effect distillation (MED) for seawater desalination process, using titanium tubes enhanced with polymer (polyethylene (PE)-expanded graphite (EG)) composite were conducted by Tahir et al. [128]. In the study, they discovered that MED built on polymer composite tubes preferred economic and carbon pollution indicators, with the ability to reduce the cost of the MED evaporator by 40% less than the cost of the titanium evaporator.

## 6. Drawbacks and Challenges

According to a review of polymer composites filled with metal derivatives, it is clear that the metal filled polymer composites are favorable materials in terms of thermal and fire retardance properties and have a great potential in fire safety applications. Yet, the enhancements are far from what is needed for various fire safety applications. In this manner, numerous technical barriers such as dispersion of fillers within their matrix, structural control, contact between individual fillers, and interfacial interaction filler/matrix should be considered to realize the wide applications of these advanced composites [129].

The poor interfacial adhesion between metal filler and polymeric matrix has been the essential factors in designing and fabricating high flame retardance performance composites. This is due to most metallic derivatives that are incompatible with most organic polymers and the number of residual groups on the filler surface is still insufficient to produce strong adhesion with the polymer matrix. Metal compounds as the inorganic components show catalytic effect on reducing smoke emission and promote the char forming process. Thus, the hybridizing of metal/polymer composites in a feasible manner would result in significant advantages to promote these characteristics, which represent intriguing characteristics superior to normal FRs [130]. For instance, the good compatibility of metal oxides reinforced polyurethane composite exhibited superior pHRR reduction and LOI value as compared to normal FRs [90].

Good dispersion and orientation of metal derivative fillers in polymers for fire retardant applications are also fundamental challenges. In some cases, the metal fillers that are in uniaxial alignment inside polymer matrix would reduce dispersivity of the fillers well. Besides that, the application of metallic fillers in polymer composites would limit their dispersivity, due to the presence of abundant impurities and residual groups on the fillers’ surface. This will increase the interlayer spacing and decrease the van der Waals interaction between the metallic fillers and polymer resins [131].

Whether metal derivative fillers can have a better impact on thermal and flame retardance properties than common FRs remains an open question for polymer-based composites. Nevertheless, common FRs may not compete with metal derivative fillers in terms of cost since a huge amount of filler volume is needed for composite applications. The structure of FRs composites and appropriate manufacturing technique has to be developed to ensure an optimized used of metal fillers and to obtain high performance flame retardance composites.

## 7. Conclusions and Future Outlooks

This review discusses various types of metal components of FRs with different types of polymer, such as LDPE, PP, rubber, and PA. The incorporation of metal to polymer composite systems has ushered in a modern age in polymer composites for a variety of applications. Different types of metal components with distinct and special properties have promising strong electrical and thermal conductivity features as polymer matrix reinforcements. One of the most notable discoveries of this review is that metal components efficiently enhance the flame retardancy of polymer composites. The use of qualitative case studies is a well-established approach for determining the chemical nature of FRs, which includes metal oxides and hydroxides, boron-based, halogen-based, phosphorus-based, synergistic, and intumescent. Metal components are combined with FR’s chemical nature to strengthen the thermal properties and flammability of polymers. Moreover, different compositions of metal components and chemical nature of FRs prove that self-reinforced composite properties can be modified to achieve better properties. The incorporation of metal components into polymer composites has been shown to significantly improve the fire resistance, providing an insulation layer, decreased flammability, and increased tensile strength. Initially, several metal components added to FR’s chemical nature do not exhibit any improvement during the CONE test, TGA, SEM, and EDS. However, with the right ratio of material compositions, the properties of the reinforced composites exhibit better improvement than the original composites. Therefore, finding the compatible ratio of the components is significant in this experiment. Most metal components used to improve fire retardancy positively affect the reinforced composites. According to the articles reviewed in this study, a positive potential outlook for research on the integration and functionalization of polymer with metal components for a new generation of high-performance composites can be expected and could have a bright future. Although there is no doubt that metal filled polymer composites can promote pioneering science and lead to industrial advancements. In recommendation, additional fundamental studies are needed to gain a deeper understanding of the relationship between metal components in this rapidly growing class of polymer composite materials. The crucial understanding and characterization of each metal in these advanced polymer composites, as well as experimental and theoretical proofs, is needed to make a prediction on the overall metal-polymer interaction. Based on the latest applications, metal-polymer hybridization has the ability to be used in a variety of fields, including aerospace, sport, electronic, and computer applications.

## Figures and Tables

**Table 1:** Various metallic fillers use for fire retardant applications [69].

Flame Retardant Chemical Nature	Example of Flame Retardants	Working Mechanism
Metal oxides and hydroxide	Magnesium hydroxide, Aluminum hydroxide, alumina trihydrate, calcium carbonate	Heat sink
Boron based	Boric acid, borax, Zink borate, boron phosphate	By forming the insulating layer
Halogen based	TCPA, TBPA, Polybrominated diphenyl ethers, Polybrominated biphenyl	Gas-phase
Phosphorus based	THPC	Condense phase
Synergistic	P/N, Halogen/Antimony tri-oxide, P/halogen	The presence of other compounds would increase the slowness of the flame emitted by the major compound.
Intumescent	Acid donor (ex-phosphoric acid, ammonium polyphosphate), carbonizing agent (ex-pentaerythritol), bowling agent (ex-melamine, urea)	Both in the gas and condensed phase

**Table 2:** Reported type for metal components of flame retardant on different types of composites.

Metal Components	Composites	Effect of Reinforcement	Reference
Metal hydroxide	Ethylene-vinyl acetate (EVA)	Form new layer that acts as insulation to flame	[88]
Silicon-containing, metal hydrate and oxide	Polypropylene (PP)	Decreasing the flow rate of the burning surface	[89]
Metal oxides	Thermoplastic polyurethane (TPU)	Low flammability and smoke emission	[90]
Zinc borate and magnesium hydroxide	Sawdust/rice husk filled polypropylene	A marginal reduction in mechanical properties and reduce flammability	[91]
Magnesium hydroxide (Mg(OH)_2_ and zinc borate (Zb)	Fiber/polypropylene	Improved thermal stability and flame retardancy	[92]
Magnesium hydroxide	Ethylene-vinyl acetate (EVA)	Better water resistance, flame retardancy, and higher pyrolysis temperature	[93]
Salicylaldoxime and chelated copper(II)salicylaldehyde	Polyethylene (PE)	Provide good flame retardant behavior	[94]
Metal chelates	Polyvinyl alcohol (PVA)	Promotes thermal stability and improve flame-retardant	[95]
Aluminum and magnesium hydroxides	Rubbers and ethylene-vinyl acetate (EVA)	No corrosive or potentially toxic substances occur and reducing the smoke level	[96]
Zinc phosphonate	Glass-fiber reinforced poly(butylene terephthalate)	No improvement on fire behavior satisfactorily	[97]
Manganese (IV) oxide (MnO_2_), zinc oxide (ZnO), and nickel(III) oxide (Ni_2_O_3_)	Polypropylene (PP)	Enhance the charring and corresponds well to the gas release with increasing temperature	[98]
Magnesium hydroxide and alumina trihydrate	Low-density polyethylene (LDPE) and ethylene-vinyl acetate (EVA)	Superior thermal stability and reduction of gases produced during burning	[99]
Nanometer titanium dioxide (nano-TiO_2_), aluminum oxide (Al_2_O_3_), and magnesium aluminate spinel (MgAl_2_O_4_)	Ammonium polyphosphate-pentaerythritol-melamine (APP-PER-MEL)	Enhance fire-resistant and anti-aging properties of the APP-PER-MEL coating	[100]
Ionic liquid-based metal-organic hybrid (PMAIL)	Epoxy resin (EP)	Total smoke production was reduced	[101]
Aluminum phosphonate (AlPi), antimony oxide, and nanometric iron oxide	Poly(3-hydroxy-butyrate-co-3-hydroxyvalerate) /poly(butylene adipate-co-terephthalate) (PHBV/PBAT)	Great pyrolysis and the fire retardancy	[102]
Aluminum trihydrate	Ethylene-vinyl acetate (EVA) and montmorillonites (MMT)	Improvement of thermal stability and flame retardancy	[103]
Iron, magnesium, aluminum, and zinc	Paraffin	Increase the char yield and decrease volatilization for the combustible gases	[79]
Metal hydroxides and antimony trioxide	Thermoplastics	Improvements in thermal stability and pigmentation properties	[104]
Metal-based organic (MBO)	Polyvinylchloride (PVC)	Improved resistance to ignition, flame spread, and smoke generation	[105]
Aluminum trihydrate	Ethylene-vinyl acetate (EVA)	Reduction in heat release rates	[106]
Silicon-containing materials and metal oxides	Aliphatic and aromatic phosphonates	Good smoke suppressant effects	[107]
Zinc borate (ZnB)	Polyamides, polyesters, polyolefin, and boron Compounds	Lower heat release and lower total heat evolved	[108]
Metal Phosphonates and Aluminum Oxide Hydroxide	Polyamide, Polyesters, and phosphorous	Improved flame-retardant and mechanical or electrical performance	[109]
Aluminum hydroxide (Al(OH)_3_)	Cycloaliphatic polyamine, epoxy resins	Small burned area and better tensile strength properties	[110]
Metal chelates, chromium acetylacetonate, and zinc acetylacetonate	Polypropylene and poly(4,4-diamino diphenyl methane-O-bicyclicpentaerythritol phosphate-phosphate)	A denser char layer was established on the composite	[111]
Metal hydroxides	Silicon	Improve the thermal protective layer build on the polymer’s surface	[78]
Alumina trihydrate, montmorillonite (MMT)	Ethylene-vinyl acetate and nanocomposite	Improve the fiber-matrix adhesion	[112]
Zinc borate, and magnesium hydroxide (Mg(OH)_2_)	Polypropylene and ammonium polyphosphate	Thermal stability and fire retardancy were improved	[92]
Titanium dioxide	Ammonium polyphosphate-pentaerythritol-melamine (APP-PER-MEL)	Anti-aging properties of the flame-retardant coating were improved	[100]
Melamine poly (zinc phosphate) (MPZnP)	Epoxy resin (EP) and polyphosphate	Earlier decomposition and slightly changed evolved gas	[113]
Metallic oxide and Metal hydroxide	Graphene foam	Better flame retardant and compressible structure	[114]
Manganese and metal salts	Ammonium polyphosphate and cellulose	Enhancing flame retardant efficiency	[115]
Zinc hydroxyl stannate and alumina trihydrate	Ethylene-vinyl acetate, polyurethane, styrene-butadiene rubber, silicone rubber, and polychloroprene rubber.	Improvement of fire resistance and better mechanical and thermal properties of the elastomer	[116]
Ammonium bromide, manganese(II), iron(II), calcium, zinc oxalate, and metal oxalates	Polyamide and cotton	Reduction of combustion rate for cotton	[117]
Nickel-metal hydride, nickel-cadmium (Ni-Cd), and metal oxide	Graphites	Excellent ability for flame-retardance, cell performance, and wettability improvement	[118]
Copper metal complex	Polyurethane	Superior flame retardant and antimicrobial properties	[119]
Diphenyl phosphates and calcium hypophosphite,	Polycarbonates and polyurethanes	Good thermal stability and low volatility	[120]
Metal hydroxides, metal hydrate, and alumina trihydrate	Ethylene-vinyl acetate and octadecylamine	Improvement of tensile and flame-resistance properties	[121]
Cupric and zinc ions	Polyethylenimine and ramie fabric	Improved thermal stability and reduced flammability	[122]
Zinc Borate and metal hydroxide	Polyethylene terephthalate, woven and organophosphorus	Decrease smoke release but no flammability improvement	[123]

## Data Availability

No new data were created or analyzed in this study. Data sharing is not applicable to this article.
